# Physical function and cognition in older adult women: the Study of Women’s Health Across the Nation (SWAN)

**DOI:** 10.1002/alz.086153

**Published:** 2025-01-03

**Authors:** Jillian Baker, Lauren MacConnachie, Michelle Hood, Carol A. Derby, Bradley M. Appelhans, Alexis Reeves, Carrie A. Karvonen‐Gutierrez

**Affiliations:** ^1^ University of Michigan, Ann Arbor, MI USA; ^2^ Department of Neurology, and Department of Epidemiology and Population Health, Albert Einstein College of Medicine, Bronx, NY USA; ^3^ Rush University, Chicago, IL USA; ^4^ Stanford University, Stanford, CA USA

## Abstract

**Background:**

Menopause is a time of accelerated loss of physical function, illustrated by challenges to mobility, speed, strength, and performance of activities of daily living. Physical function is associated with cognitive function, but there are limited data exploring this association among older women. In a cohort of older adult women, we hypothesize better performance on measures of physical function will be associated with better cognitive performance. This paper aims to characterize the link between physical function and cognition in older women, which may suggest interventions to support health in both domains of function.

**Methods:**

The Study of Women’s Health Across the Nation (SWAN) is a multi‐ethnic, community‐based study of women followed through and beyond the menopausal transition. At Visit 15 (V15, 2015‐16), SWAN participants completed self‐reported measures (SF‐36) and performance‐based measures (gait speed and stair climb) of physical function and tests of cognition, including symbol digit modalities test [SDMT], East Boston Memory Test‐Delayed and ‐Immediate (EBMT‐D/EBMT‐I), and digit span backwards (DSB). A global cognitive z‐score was calculated by averaging the z‐scores of the four cognitive tests.

In separate linear regressions with cross‐sectional (V15), we regressed SDMT and global z‐score on each measure of physical function (SF‐36, gait speed, stair climb), adjusting for demographic factors, socioeconomic status (SES), and health conditions.

**Results:**

Our sample included 1703 women who had at least one measure of physical function and cognition at V15, with a mean age of 65.5 years (SD = 2.69); 47.6% were white, 26.8% Black, 10.2% Chinese, 9.9% Japanese, and 5.5% Hispanic. Almost half (48.9%) attained a college degree. All but one woman were post‐menopausal, indicating at least 12 months elapsed since the final menstrual period.

Higher SF‐36 scores and faster gait speeds were associated with faster processing speed (SDMT, β = 0.04, p = 0.005; β = 2.69, p = 0.039, respectively) and global cognition (β = 0.002, p = 0.037; β = 0.23, p = 0.006, respectively). Slower stair climbs were associated with worse processing speed (SDMT β = ‐0.18, p<0.001) and global cognition (β = ‐0.01, p<0.001).

**Conclusions:**

In cross‐sectional analyses in SWAN, physical functioning is positively associated with cognition among older adult women. Future analyses with longitudinal data are needed to assess whether better physical performance predicts better cognition as women age.

